# Disentangling Race, Poverty, and Place in Disparities in Physical Activity

**DOI:** 10.3390/ijerph16071193

**Published:** 2019-04-03

**Authors:** Armani M. Hawes, Genee S. Smith, Emma McGinty, Caryn Bell, Kelly Bower, Thomas A. LaVeist, Darrell J. Gaskin, Roland J. Thorpe

**Affiliations:** 1 Hopkins Center for Health Disparities Solutions, Johns Hopkins Bloomberg School of Public Health, Baltimore, MD 21205, USA; armani.hawes@gmail.com (A.M.H.); genee.smith@jhu.edu (G.S.S.); cbell7@umd.edu (C.B.); kbower1@jhu.edu (K.B.); TAL@tulane.edu (T.A.L.); dgaskin1@jhu.edu (D.J.G.); 2 Department of Environmental Health and Engineering, Johns Hopkins Bloomberg School of Public Health, Baltimore, MD 21205, USA; 3 Department of Health Policy and Management, Johns Hopkins Bloomberg School of Public Health, Baltimore, MD 21205, USA; emcgint1@jhu.edu; 4 Johns Hopkins School of Nursing, Baltimore, MD 21205, USA; 5 Department of Health Policy and Management, Tulane University School of Public Health and Tropical Medicine, New Orleans, LA 70112, USA; 6 Department of Health, Behavior, and Society, Johns Hopkins Bloomberg School of Public Health, Baltimore, MD 21205, USA

**Keywords:** segregation, race, physical activity, disparities

## Abstract

Significant racial disparities in physical activity—a key protective health factor against obesity and cardiovascular disease—exist in the United States. Using data from the 1999–2004 National Health and Nutrition Examination Survey and the 2000 United States (US) Census, we estimated the impact of race, individual-level poverty, neighborhood-level poverty, and neighborhood racial composition on the odds of being physically active for 19,678 adults. Compared to whites, blacks had lower odds of being physically active. Individual poverty and neighborhood poverty were associated with decreased odds of being physically active among both whites and blacks. These findings underscore the importance of social context in understanding racial disparities in physical activity and suggest the need for future research to determine specific elements of the social context that drive disparities.

## 1. Introduction

Physical activity is a major protective factor for several health outcomes, including obesity, coronary heart disease, stroke, and hypertension [1,2]. In 2010, 58.4% of non-Hispanic blacks failed to meet physical activity guidelines, compared to 45.1% of non-Hispanic whites [3] (Schiller JS et al. 2012). Although differences in physical activity across races and ethnicities are well documented, little is known about the nature of these disparities. Previous research has examined factors that may explain racial disparities in physical activity prevalence [4,5], including a combination of individual risk factors such as air pollution, lower access to recreation facilities, and a less safe environment [6,7,8,9,10]. Blacks and whites tend to live in very different environments, and racial disparities in physical activity prevalence likely result in part from differences in health risk exposures and/or healthcare resources related to poverty and place [6,11]. 

Residential segregation is the physical separation of two or more groups into different neighborhoods. Thus, segregation can lead to very different living environments at the neighborhood level [11]. Overt laws mandating segregation are illegal in the United States, however housing patterns show significant and persistent segregation by race and income groups. In 2000, the index of dissimilarity showed that 66% of blacks in the United States would have to move to eliminate segregation [12]. This clear division of races can be attributed to discriminatory lending practices that favored whites relative to blacks. These unfair lending practices were fostered and reinforced by government housing and community development policies [13,14,15,16]. 

Racial segregation is thought to be a fundamental cause of health disparities because it can lead to vastly different environmental and social risk exposures [11,17,18,19,20,21,22,23]. However, empirical evidence demonstrating this relationship is still limited. One prior study has explored the relationship between residential segregation and physical activity, emphasizing two main pathways through which segregation impacts physical activity: resource deprivation and risk exposure [7]. However, these findings showed no significant association between residential segregation and physical activity for blacks. A more recent study came to the same conclusions [24]. However, both studies presented substantial limitations in their measurements of segregation which may have led to statistically insignificant results. Both prior studies measured segregation within metropolitan statistical area (MSA) which often includes diverse neighborhoods with significant variation in segregation status and other important factors, such as neighborhood wealth. As a result, these studies may have failed to accurately capture the relationship between residential segregation and physical activity. Therefore, it is important to further investigate this relationship using more precise measures of residential segregation and accounting for other important potentially confounding factors, such as individual and neighborhood poverty [9,25,26,27,28,29].

This work is guided by the multiple stratification perspective [30,31,32] which seeks to understand how dimensions of stratifications operate synergistically to shape one’s health or health behaviors. In this study, we are seeking to understand how three dimensions of stratification: race, poverty, and “place” impact physical activity. This study is an advancement of previous work in that it seeks to disentangle the often confounded race, concentrated poverty, and neighborhood conditions [11,33]. 

The objective of this study is to disentangle the effects of race from individual socioeconomic position, neighborhood racial composition, and neighborhood concentrated poverty. Specifically, it aims to determine the influence of race, individual-level poverty, racial composition, and neighborhood-level poverty on the prevalence of physical activity. We hypothesize that racial disparities in physical activity prevalence will be largest between blacks in primarily black neighborhoods versus whites in primarily white neighborhoods; between poor adults in poor neighborhoods versus non-poor adults in non-poor neighborhoods; and between poor blacks in poor neighborhoods versus non-poor whites in non-poor neighborhoods. 

## 2. Methods

### 2.1. Data

The National Health and Nutrition Examination Survey (NHANES) is an annual survey released in two-year increments and was designed to determine the health, functional, and nutritional status of the United States (US) population. Each sequential series of this cross-sectional survey was nationally representative of the civilian non-institutionalized US population. There was over sampling of low-income individuals, children ages 12 to 19 years, adults ages 60 years and older, African Americans, and Mexican Americans. The sampling design for each panel was a complex, stratified multistage probability sample. Data were collected from respondents in two phases. The first phase consisted of a home interview in which information regarding the participant’s health status and history, health behaviors, health services use, and behavioral risk factors were obtained. The second phase was a medical examination. At the conclusion of the home interview, participants were invited to receive a detailed physical examination at a mobile examination center. Details regarding the data collection procedure can be found elsewhere [34].

Combined data from the 1999–2004 NHANES was linked to the 2000 US Census data in order to measure neighborhood racial composition and neighborhood poverty within the respondents’ census tract of residence. Because we accessed the respondents’ census tract, the analysis was performed at the National Center for Health Statistics (NCHS) Research Data Center under the supervision of NCHS staff to preserve the privacy, confidentiality, and anonymity of the NHANES respondents. The Institutional Review Board at the Johns Hopkins Bloomberg School of Public Health approved the study protocol under IRB Exemption 4 for the protection of the study participants. We restricted the analysis to 7572 non-Hispanic African Americans/blacks and 12,106 non-Hispanic whites who were aged 25 and older.

### 2.2. Measures

#### 2.2.1. Physical Activity Measurement

We used two questions to derive our physical activity measure. The first question was based on participants’ report of tasks inside or outside the home, including the yard, for at least 10 minutes that caused light sweating or a slight to moderate increase in heart rate or breathing in the past month [35]. The second question was based on the participants’ report of vigorous activities for at least 10 minutes that caused heavy sweating, or a large increase in breathing or heart rate in the past month [35]. Those who reported yes to both questions were considered to be physically active. 

#### 2.2.2. Key Independent Variables Measurement

The main independent variables of interest were individual race, individual poverty, neighborhood racial composition, and neighborhood poverty. Individual race was self-reported in the NHANES as either non-Hispanic African American/black or non-Hispanic white (hereafter referred to as black and white). We measured individual poverty status in two ways. The poverty income ratio is a ratio of household income to the Federal Poverty Level (FPL) and is based on the respondent’s household income and size. The poverty income ratio was coded as a five-level categorical variable that indicates each individual’s household poverty ratio and ranged from below FPL to above 400 percent of FPL. This categorization was used in our race–place model. Additionally, we used a binary poverty variable in our poverty-place model to indicate whether individuals had household incomes below or above 200 percent of the FPL. 

We used the respondents’ census tracts to measure neighborhood characteristics because of their status as small and permanent statistical subdivisions within a county that range from 1500 to 8000 persons who are similar with respect to population characteristics, economic status, and living conditions. Neighborhood racial composition was designated as predominantly white, black, or Hispanic/other race (Asian, Hawaiian or Pacific Islander, or other) if that group made up greater than 65 percent of the census tract’s population. The racial composition of a neighborhood was designated as integrated if at least two groups each made up more than 35 percent of the census tract’s population. Finally, neighborhoods were classified as poor if greater than or equal to 20 percent of families in that census tract had incomes below the FPL. This measure of neighborhood poverty is not only associated with numerous health outcomes, however it is also associated with other measures of economic deprivation including deterioration in housing, unemployment, low earnings, and crime [36,37].

To disentangle the effects of individual race and neighborhood racial composition on physical activity, we created a race–place dummy variable with the following eight categories: white in white neighborhood, white in black neighborhood, white in other race neighborhood, white in integrated neighborhood, black in black neighborhood, black in white neighborhood, black in other race neighborhood, and black in integrated neighborhood. To disentangle the effects of individual poverty and neighborhood poverty on physical activity, we created a poverty–place dummy variable including: non-poor in non-poor neighborhood, poor in non-poor neighborhood, non-poor in poor neighborhood, and poor in poor neighborhood. To disentangle the effects of individual race, individual poverty, and neighborhood poverty on physical activity, we created a race–poverty–place dummy variable with the following eight categories: non-poor white in non-poor neighborhood, non-poor white in poor neighborhood, poor white in non-poor neighborhood, poor white in poor neighborhood, non-poor black in non-poor neighborhood, non-poor black in poor neighborhood, poor black in non-poor neighborhood, and poor black in poor neighborhood.

Covariates included variables that may confound the relationship between race and physical activity. These included demographic variables (age and gender), socioeconomic factors (education and health insurance status), and health factors (weight and smoking status, presence of comorbid conditions, and self-reported health). Age was measured as a continuous variable. Gender was coded as a dichotomous variable. Educational attainment was coded as four categories (<12 years of school, high school graduate/general education diploma (GED), some college, or college graduate or higher). Health insurance was coded as three categories (privately insured, publicly insured, or uninsured). We also controlled for living environment as urban or non-urban (living in an MSA or not living in an MSA).

### 2.3. Statistical Analysis

We first conducted bivariate analysis comparing the prevalence of physical activity across the race categories. We then estimated multivariate logistic models to examine the associations between individual race and poverty, neighborhood racial composition, neighborhood poverty, and physical activity. We examined the impact of potential confounders by creating a base model. The base model determined if individual covariates separately influenced the odds of being physically active. We then considered the impact of the additional independent variables. The race–place model shows whether the odds of being physically active were associated with adults’ individual race relative to the racial composition of their neighborhood compared to whites in white neighborhoods. The poverty-place model shows whether the odds of being physically active were related to adults’ poverty status relative to their neighborhood’s poverty level compared to non-poor adults in non-poor neighborhoods. Finally, the race–poverty–place model shows whether the odds of being physically active were related to adults’ individual race, individual poverty, and neighborhood poverty compared to non-poor whites in non-poor neighborhoods.

Sample weights were applied to account for the differential probability of being selected, non-response adjustments, and adjustments to national control totals in the NHANES. Taylor linearization procedures were used to account for the multi-stage sampling design factors on NHANES. We used the SVY commands in STATA 11 to produce nationally representative estimates and appropriate standard errors for all estimations. P-values less than 0.05 were considered statistically significant and all tests were two sided. Statistical analyses were conducted using Stata version 11.

## 3. Results

The distribution of characteristics of the study population by race is shown in Table 1. Compared to whites, blacks were younger, more likely to be below the poverty threshold, and more likely to live in urban areas. Blacks also had lower levels of education and were more likely to have public insurance, be obese, and be current smokers. Blacks also self-rated their health as worse than whites. Of the 7572 blacks, 28.2% were physically active, while 53% of the 12,106 whites were physically active. The prevalence of physical activity varied with key independent variables and covariates. 

The association between individual race and physical activity is displayed in Table 2. We found that age, gender, urban location, education, health insurance, weight status, comorbid conditions, smoking status, and fair/poor health were significant predictors of physical activity. In the base model, which we adjusted for household poverty, education, insurance, gender, weight status, smoking status, self-rated health, comorbid conditions, and living in an urban environment, blacks had 54% lower odds of being physically active compared to whites (OR = 0.46; 95% CI = 0.37–0.58). The prevalence of physical activity was inversely related to household poverty level. Adults in poor households (below FPL) had the lowest odds of being physically active (OR = 0.31; 95% CI = 0.20, 0.48), followed by adults 100–199% below FPL (OR = 0.48; 95% CI = 0.36–0.66) and then adults between 200% and 299% FPL (OR = 0.57; 95% CI = 0.39–0.82).

The race–place model tested whether the odds of being physically active was related to adults’ racial identity relative to the racial composition of their neighborhood (See Table 2). In this model, gender, age, living in an urban location, education level, insurance status, and smoking status were still significant predictors and were similar in magnitude to the base model. Blacks in integrated and black neighborhoods had significantly lower odds of being physically active than whites in white neighborhoods (OR = 0.40; 95% CI = 0.29–0.55 and OR = 0.60; 95% CI = 0.37–0.97, respectively). Additionally, blacks in white neighborhoods had significantly lower odds of being physically active (OR = 0.58; 95% CI = 0.42–0.80). Whites in integrated neighborhoods had significantly lower odds of being physically active than whites in white neighborhoods (OR = 0.68; 95% CI = 0.51–0.91).

The results from the poverty–place model tested whether the odds of being physically active were related to adults’ poverty status relative to the poverty of their neighborhood (Table 2). We found that poor adults in poor neighborhoods had the lowest odds of being physically active compared to non-poor adults in non-poor neighborhoods (OR = 0.26; 95% CI = 0.18–0.38). Poor adults in non-poor neighborhoods (OR 0.55; 95% CI 0.41–0.70) and non-poor adults in poor neighborhoods (OR 0.57; 95% CI 0.39–0.84) also had significantly lower odds of being physically active compared to non-poor adults in non-poor neighborhoods. Individual and neighborhood poverty appear to play an important role in physical activity for both whites and blacks. Additionally, individual race remained significant in this model. The odds of being physically active were 46% (95% CI = 0.43–0.69) lower for blacks compared to whites.

Finally, the race–poverty–place model examined the odds of being physically active with regard to individual race, individual poverty, and neighborhood poverty (See Table 3). This showed us three main findings. First, we saw a poverty–place gradient for poor blacks and poor whites. Compared to non-poor whites in non-poor neighborhoods, poor blacks and poor whites living in poor neighborhoods were least likely to be physically active (OR = 0.21; 95% CI = 0.13–0.35 and OR = 0.21; 95% CI = 0.11–0.39, respectively). Individual and neighborhood poverty appear to play a more important role in physical activity than race. Finally, the overall trends suggest that there is a place gradient for blacks and poor whites. Compared to non-poor whites in non-poor neighborhoods, the odds of being physically active for blacks and poor whites are lower when they live in poor neighborhoods. To make this claim, we saw that the odds of being physically active for poor blacks was significantly lowered when living in poor neighborhoods as compared to non-poor neighborhoods (OR = 0.21; 95% CI = 0.12–0.33 and OR = 0.27; 95% CI = 0.17–0.43, respectively). We found similar changes when comparing poor whites in poor and non-poor neighborhoods (OR = 0.21; 95% CI = 0.11–0.39 and OR = 0.55; 95% CI = 0.41–0.75, respectively). Although the confidence intervals overlap, the overall trends suggest that there is a place gradient for poor whites and blacks.

## 4. Discussion

In this study, we sought to disentangle the effects of race, individual poverty, neighborhood poverty, and residential segregation on physical activity prevalence. We found that each individually influenced the prevalence of physical activity. Living in racially segregated and integrated neighborhoods was associated with lower odds of being physically active for blacks. Living in poor neighborhoods was associated with decreased odds of being physically active for blacks and poor whites, however not for non-poor whites. The lack of significance for non-poor whites in poor neighborhoods may be attributed to the small sample size which augmented the confidence interval. Blacks and poor whites had lower odds of being physically active compared to non-poor whites. Underlying these findings is that both individual poverty and neighborhood poverty appear to make the biggest difference on disparities in physical activity, as opposed to individual race having the most influence.

We found that, compared to whites in white neighborhoods, blacks had decreased odds of being physically active regardless of neighborhood racial composition. Our findings were somewhat consistent with our hypothesis that the odds of being physically active would be lowest for blacks in black communities. In addition, black and whites in integrated neighborhoods had the lowest odds of being physically active. An explanation for these findings is that blacks and whites are living in similar social conditions which influence their ability to engage in physical activity. For example, it is possible that the conditions might be inadequate for engagement in physical activity [38] or infrastructure in the neighborhoods to encourage physical activity [11,39]. This is consistent with the Exploring Health Disparities in Integrated Communities Study, which reported that poor blacks and whites living in integrated communities of equally low socioeconomic conditions had similar rates of physical inactivity [40]. Our finding that segregation for blacks was associated with lower odds of being physically active is not consistent with two earlier studies which found no association between segregation and physical activity [7,24]. By measuring segregation with a smaller geographic unity, the US census tract, instead of MSA, we believe that we provide a more nuanced examination of the relationship. Census tracts are substantially more sensitive and predictive measures of segregation because they typically represent neighborhoods and population characteristics, while MSAs represent larger areas [36]. Therefore, census tracts are able to capture certain living elements which influence health behaviors and outcomes that MSAs tend to overlook [41].

Furthermore, we found that neighborhood poverty appears to influence physical activity, perhaps to a greater extent than individual poverty. These findings are evidenced by lower odds of physical activity among both poor and non-poor individuals in poor neighborhoods, supporting our hypothesis that racial disparities would be largest between poor adults in poor neighborhoods and non-poor adults in non-poor neighborhoods. These results are consistent with those of the Moving to Opportunity (MTO) demonstration project, which found that enabling families to move from high poverty to lower poverty neighborhoods resulted in improved health behaviors and outcomes [42]. Poor neighborhoods may have contributed to lower physical activity prevalence in blacks and poor whites due to the many barriers to exercise, such as access to parks and recreational facilities, air pollution, unsafe conditions, and a poor physical layout and design of a city [9,43].

Finally, our findings support our hypothesis that it is not race or individual poverty or neighborhood poverty alone that contributes to disparities, however, rather, it is a combination of these factors [33,44]. Moreover, our findings suggest that individual and neighborhood poverty are more significant predictors of low physical activity than race. These results are consistent with previous research which found that place is important in determining health behaviors and outcomes [11]. This consistency is important in recognizing that race alone is not responsible for disparities. Rather, they are largely driven by barriers in socioeconomic environments, emphasizing the importance of community level risk factors [45].

Our study is among the first to investigate and disentangle the effects of race, poverty, and place on physical activity using a nationally representative sample. By defining neighborhood racial composition at the census tract level, we were able to depict a more accurate picture of neighborhood racial composition. Although our more sensitive measure of segregation is a unique and important element of our study, a stronger measure may have been more accurate. For instance, instead of measuring segregation from census tracts, future studies may consider examining the racial composition of census tracts relative to a surrounding area. By understanding how a neighborhood compares to its surroundings, we may capture a more accurate representation of how segregation impacts health behaviors and outcomes. In addition, there are other measures of segregation including concentration, clustering, centralization, and hypersegregation that should be carefully examined in future work. Examining multiple dimensions of segregation can provide insight into implications for understanding race differences in physical activity.

There are additional limitations to this study. Because it was a cross sectional analysis, our results cannot claim causality. Further, our findings are generalizable only to blacks and whites. Future work will need larger sample sizes if they wish to consider Hispanics, Asians, Native Americans, or other races and ethnicities. Also, the study pooled five (1999–2004) years of data from the NHANES to obtain adequate sample sizes, requiring the assumption that associations remained stable over that period. By using the 2000 US Census data to measure neighborhood racial composition and neighborhood-level poverty, we assumed that these measures remained stable in the census tract throughout the study period. An equally important weakness involves the measurement of physical activity. Because we measured physical activity as a single-item assessing any exercise in the past month, there is some concern for its validity. Furthermore, our measure represents a combination of work-related physical activity and leisure time physical activity. One study found weak correlations between a single-item measure of physical activity and physical activity measured by accelerometer [46]. Another study found a significant correlation between self-reported physical activity and pedometer values, showing Spearman’s rho of 0.607 and a *p*-value of 0.003 [47]. A previous report confirmed that self-reported physical activity is suitable for studying physical activity prevalence [48]. Future studies measuring physical activity should consider examining work-related physical activity and leisure time physical activity separately. In spite of these weaknesses, our study is unique because it is the only known study to examine the effects of race, poverty, and place on physical activity disparities in a nationally representative sample.

Because individual and neighborhood poverty matters for blacks and whites, policies must address barriers faced by poor individuals and poor neighborhoods. Likewise, as the results from our race–place model suggest, racial integration of neighborhoods alone will not improve physical activity prevalence, so efforts that look past racial lines are crucial. Because the relationship between the built environment of neighborhoods and physical activity depends on socioeconomic context, certain neighborhood development policies may encourage physical activity. For instance, to enhance the built environment, policies should increase the number of parks and recreational facilities and improve the quality of sidewalks and neighborhood walkability [49]. Urban planning policies also have immense potential to transform poor neighborhoods and connect them to opportunities. One idea is Transit-Oriented Development (TOD), which is a high-density, mixed-use residential area with access to ample amounts of transportation. TOD has been shown to improve mobility choices, increase households’ disposable income, reduce air pollution, assist in more affordable housing, and revitalize declining urban neighborhoods [50]. Similarly, policies involving mixed-income housing could help lift neighborhoods out of poverty. The previously mentioned MTO project has been a successful endeavor encouraging mixed-income neighborhoods, which can increase neighborhood safety and, thus, encourage physical activity [42,51]. Likewise, because crime and physical activity rates have been found to be negatively associated, successful gun violence prevention and drug policing policies may encourage physical activity [52,53,54,55]. Because low income status disproportionately affects racial minorities, these types of initiatives can help reduce racial disparities [11].

## 5. Conclusions

By achieving a better view of how segregation influences the relationship between race and physical activity, we were able to disentangle its effects from individual and neighborhood-level poverty. When interpreting the findings of the current study, it is important to recognize the potential for real world applications. Poverty is the most important factor influencing an individual’s physical activity level; however, ethnicity (race) and the culture of the community and the opportunities in the area in which you live are also crucially important factors. We urge researchers and policymakers to invest in new ways of dealing with disparities in physical activity and promoting a healthier lifestyle. Any public health interventions will first require the foundation of a strong shift in policies that better address problems faced by the poor. Although it appears to be an exceptionally multifaceted task, we anticipate results from this study and others will contribute to meaningful reductions in racial disparities in physical activity and promote healthy behaviors to achieve a deeper understanding of health outcomes and a better quality of life.

## Figures and Tables

**Table 1:** Characteristics of the study population of 19,678 adults, National Health and Nutrition Examination Survey (NHANES) 1999–2004.

Variable	Black *N* = 7572	White *N* = 12,106	*p*-Value
Age (mean ± SE)	46.4 ± 0.4	50.2 ± 0.3	<0.001
Male, %	43.8	48.2	<0.001
Individual Poverty, %			<0.001
Above Poverty Thresh(PIR 4+)	21.7	43.7	<0.001
Above Poverty Thresh(PIR 3–3.99)	11.2	14.8	0.012
Above Poverty Thresh(PIR 2–2.99)	17.2	16.1	0.532
Above Poverty Thresh(PIR 1–1.99)	26.2	17.4	<0.001
Below Poverty Thresh(PIR < 1)	23.8	7.9	<0.001
Urban, %	91.6	80.9	<0.001
Education Level, %			<0.001
<12th grade	34.6	14.1	<0.001
High School Grad/GED *	24.0	27.6	<0.001
Some College	27.5	29.5	<0.001
College Grad	14.0	28.8	<0.001
Health Insurance, %			<0.001
Private	58.4	73.5	<0.001
Public	20.7	15.3	<0.001
No Insurance	20.9	11.2	<0.001
Comorbid Conditions, %	1.81	1.70	0.012
Weight Status, %			<0.001
Normal	25.4	34.4	<0.001
Overweight	37.4	39.7	<0.001
Obese	37.2	25.9	<0.001
Smoking Status, %			<0.001
Current Smoker	27.6	23.3	0.192
Former Smoker	17.2	29.6	<0.001
Never Smoked	55.2	47.1	<0.001
Self-Rated Health as Fair/Poor, %	25.8	14.7	<0.001
Physically Active, %	28.2	53.0	<0.001

* GED stands for general education diploma.

**Table 2:** Estimated odds of being physically active controlling for the race, neighborhood-level poverty, and racial composition of the neighborhood and the race–racial composition of the neighborhood.

Variable	Base Model		Race–Place Model	
	Odds Ratio	95% CI	Odds Ratio	95% CI
Black	0.46	0.37–0.58	-	-
Neighborhood level poverty	-	-		
Predominately white neighborhood	-	-	-	-
Predominately black neighborhood	-	-	-	-
Predominately other race neighborhood	-	-	-	-
Integrated neighborhood	-	-	-	-
White in white neighborhood	-	-	Ref	Ref
White in black neighborhood	-	-	1.31	0.14–12.0
White in other race neighborhood	-	-	0.84	0.45–1.57
White in integrated neighborhood	-	-	0.68	0.51–0.91
Black in black neighborhood	-	-	0.60	0.37–0.97
Black in white neighborhood	-	-	0.58	0.42–0.80
Black in other race neighborhood	-	-	0.31	0.09–1.01
Black in integrated neighborhood	-	-	0.40	0.29–0.55
Household poverty 4 or higher	Ref	Ref	Ref	Ref
Household poverty 3–3.99 Federal Poverty Level (FPL)	0.77	0.55–1.06	0.75	0.54–1.04
House hold poverty 2–2.99 FPL	0.57	0.39–0.82	0.53	0.36–0.77
Household poverty 1–1.99 FPL	0.48	0.36–0.66	0.44	0.32–0.60
Household poverty below FPL	0.31	0.20–0.48	0.29	0.19–0.45
Urban	1.58	1.19–2.13	0.80	0.60–1.08
Less than 12th grade	Ref	Ref	Ref	Ref
High School Grad/GED	1.61	1.20–2.15	1.50	1.12–2.01
Some College	1.82	1.36–2.42	1.74	1.31–2.31
College graduate or higher	2.66	1.97–3.59	2.44	1.81–3.28
Private insurance	Ref	Ref	Ref	Ref
Public Insurance	0.62	0.50–0.76	0.60	0.48–0.75
Uninsured	0.68	0.52–0.88	0.70	0.54–0.92
Male	2.05	1.67–2.51	2.07	1.67–2.56
Normal weight	Ref	Ref	Ref	Ref
Overweight	0.99	0.79–1.23	0.98	0.78–1.24
Obese	0.68	0.54–0.86	0.70	0.55–0.89
Comorbid Conditions	1.41	0.77–2.62	1.40	0.76–2.55
Current Smoker	Ref	Ref	Ref	Ref
Former Smoker	1.84	1.35–2.52	1.79	1.31–2.46
Never Smoked	1.77	1.36–2.31	1.71	1.30–2.64
Self-Rated Health as fair/poor	0.35	0.26–0.48	0.36	0.27–0.48
Age	0.96	0.94–0.95	0.95	0.95–0.96

**Table 3:** Estimated odds of being physically active controlling for poverty–place and race–poverty–place.

Variable	Poverty–Place Model		Race–Poverty–Place Model	
	Odds Ratio	95% CI	Odds Ratio	95% CI
Black	0.54	0.43–0.69	-	-
Non-poor in non-poor neighborhood	Ref	Ref	-	-
Poor in non-poor neighborhood	0.55	0.41–0.70	-	-
Non-poor in poor neighborhood	0.57	0.39–0.84	-	-
Poor in poor neighborhood	0.26	0.18–0.38	-	-
Non-poor white in non-poor neighborhood	-	-	Ref	Ref
Non-poor white in poor neighborhood	-	-	0.66	0.38–1.15
Poor white in non-poor neighborhood	-	-	0.55	0.41–0.75
Poor white in poor neighborhood			0.21	0.11–0.39
Non-poor black in non-poor neighborhood	-	-	0.51	0.39–0.68
Non-poor black in poor neighborhood	-	-	0.35	0.22–0.55
Poor black in non-poor neighborhood	-	-	0.27	0.17–0.43
Poor black in poor neighborhood	-	-	0.21	0.12–0.33
Urban	0.78	0.59–1.03	0.80	0.13–0.35
Less than 12th grade	Ref	Ref	Ref	Ref
High School Grad/GED	1.53	1.15–2.03	1.52	1.14–2.03
Some College	1.82	1.37–2.41	1.81	1.35–2.42
College graduate or higher	2.83	2.12–3.77	2.80	2.10–3.74
Private insurance	Ref	Ref	Ref	Ref
Public insurance	0.54	0.43–0.68	0.54	0.43–0.68
Uninsured	0.65	0.50–0.85	0.66	0.51–0.86
Male	2.09	1.71–2.56	2.10	1.71–2.59
Normal weight	Ref	Ref	Ref	Ref
Overweight	0.99	0.79–1.23	0.99	0.79–1.24
Obese	0.68	0.53–0.88	0.69	0.54–0.89
Comorbid Conditions	1.34	0.75–2.39	1.35	0.75–2.43
Current Smoker	Ref	Ref	Ref	Ref
Former Smoker	1.90	1.38–2.60	1.90	1.38–2.61
Never Smoked	1.79	1.37–2.35	1.80	1.37–2.37
Self-Rated Health as fair/poor	0.36	0.27–0.48		
Age	0.96	0.95–0.96	0.95	0.95–0.96
